# Proton-pump inhibitor-induced bone loss is preventable by concomitant use of a long-acting somatostatin analogue

**DOI:** 10.22038/IJBMS.2023.71245.15571

**Published:** 2024

**Authors:** Farhad Koohpeyma, Samaneh Taghiyan, Mesbah Shams

**Affiliations:** 1 Endocrine and Metabolism Research Center, Shiraz University of Medical Science, Shiraz, Iran

**Keywords:** Bone density, Octreotide, Osteoporosis, Pantoprazole, Parathyroid hormone

## Abstract

**Objective(s):**

Long-term consumption of pump inhibitors causes osteoporosis. Some possible mechanisms are gastrin over-secretion and hypochlorhydria. Octreotide is a somatostatin analog that inhibits the secretion of many hormones such as gastrin. This study aimed to assess the effects of pantoprazole on the bone when used with octreotide in an animal model.

**Materials and Methods:**

Forty-eight male Wistar rats were randomly assigned into 4 groups: A) pantoprazole 3 mg/Kg/day orally; B) Sandostatin LAR 1 mg/month intramuscular injection; C) Pantoprazole and Sandostatin LAR; and D) Control group. After 90 days of the experiment, bone densitometry was done and serum and urine samples were collected for analysis.

**Results:**

The results indicated a significant decrease in the global, spine, femur, and tibia bone mineral density (BMD) and bone mineral content (BMC) in the pantoprazole group compared to the control group (*P*<0.05). There was a significant increase in the levels of PTH, gastrin, and alkaline phosphatase (ALP) in the pantoprazole group compared to the control group (*P*<0.05). There was no significant difference in the serum levels of gastrin, PTH, ALP, and also BMD in the rats that received sandostatin+ pantoprazole or sandostatin alone, compared to the control group.

**Conclusion:**

This study showed that the pantoprazole-induced bone loss, through elevation of serum gastrin and PTH, was preventable by concomitant use of a long-acting somatostatin analog.

## Introduction

Osteoporosis is a multifactorial disorder that causes over 8.9 million fractures annually (1). Proton-pump inhibitors (PPIs) are a group of gastric acid secretion inhibitor drugs, the long-term consumption of which may lead to osteoporosis and osteoporotic fractures (2-6). Various underlying mechanisms have been suggested for the PPIs’ effects on the bone. Some studies have revealed that gastrin over-secretion stimulates the parathyroid hormone (PTH) secretion which results in bone resorption (3, 7-9). On the other hand, hypochlorhydria of the gastrointestinal system affects calcium and magnesium absorption by reducing its ionization (10). Different studies have demonstrated that PPI consumption reduces the absorption of vitamins B6, B9, and B12 (11), and hyperhomocysteinemia which showed a negative effect on the collagenous matrix synthesis by inhibiting the lysyl oxidase enzyme (12, 13). PPIs inhibited osteoblast activity by affecting alkaline phosphatase enzyme activity in *in vitro* conditions (14). Although various investigations has been performed regarding PPIs’ effects on bone loss and osteoporosis, their exact underlying mechanism is still unknown.

Somatostatin holds an interesting place in gastrointestinal hormone balance and has originally been discovered as an inhibitor of growth hormone release (15). It is now known to inhibit a variety of gastrointestinal functions like hormone secretion including gastrin, acetylcholine, cholecystokinin, and motilin (16). The clinical use of octreotide has been established for acromegaly, secretory diarrhea, gastrointestinal bleeding, and imaging of neuroendocrine tumors (16, 17). Somatostatin analog, somatostatin LAR (Sandostatin LAR), is a slowly released formulation that requires only monthly injection and supplies high-dose, stable serum levels of octreotide (18, 19). 

Given the inhibitory effect of somatostatin on gastrin secretion and the fact that hypergastrinemia is a dominant mechanism of PPI-induced bone loss, octreotide may be able to protect the bone. This study aimed to assess the effect of pantoprazole (a PPI) on bone mineral density and various biochemical markers related to the bone with concomitant use of a long-acting somatostatin analog (Sandostatin LAR) in an animal model. 

## Materials and Methods

***Experimental animals***

Forty-eight male Wistar rats (230 ±20 g) aged three months, free of illness, were purchased from the animal laboratory of Shiraz University of Medical Sciences. The animals were maintained under a controlled setting in the animal house for 97 days (seven days for the adaptation of the animals and 90 days for the experiment). The environment temperature was kept at 22±2 °C, with an atmospheric humidity of 55% ±5% and a 12-hour light-dark cycle. The study protocol was approved by the Animal Ethics Committee of Shiraz University of Medical Sciences, Shiraz, Iran (IR.SUMS.MED.REC1399.245), and we followed the national guidelines on the care and use of laboratory animals. The experiment was conducted in accordance with the ARRIVE (Animal Research: Reporting of *in vivo* Experiments) guidelines (20) for the care and use of research animals.

***Study protocol***

All rats were randomly assigned into four groups of 12 rats as below (according to Matuszewska *et al*., 2016)(21): group A) 3 mg/Kg oral pantoprazole (Avicenna Co, Tehran, Iran) daily, group B) 1 mg/month intramuscular Sandostatin LAR Depot (octreotide acetate) (Novartis Pharma AG, Basle, Switzerland) monthly (two doses), group C) Pantoprazole & Sandostatin LAR with mentioned doses and routes of administration, and group D) the same volume of the normal saline as the drug (control group). The rats were kept in 12 cages in the same room (four rats from each group in one cage). 

***Biochemical and bone mineral density assessments***

At the end of the study, after 12 hr of fasting and under anesthesia with ketamine (10%)/ xylazine (2%) mixture (80/5 mg/kg) (Alfasan, Netherland), bone mineral density was measured and then 5 ml blood was collected by cardiac puncture. Afterward, the animals were sacrificed by sodium thiopental intraperitoneally (100 mg/kg).

Serum and urine biochemical and bone mineral densitometry of the rats were evaluated after 90 days of intervention. The serum was obtained by centrifuging blood samples for 15 min at 3000 rpm and stored at −20 °C. Serum calcium, phosphate, chloride, magnesium, alkaline phosphatase (ALP), and creatinine levels were assessed using a Biosystem kit (Biosystems SA, Barcelona, Spain). Also, serum PTH and gastrin levels were assessed with a Bioassay Technology Laboratory kit (China). The bone mineral density (BMD) and bone mineral content (BMC) of the bones were assessed by Dual X-ray absorptiometry (DXA), using a Hologic system (Discovery QDR, USA).

***Statistical analysis***

The data were analyzed using SPSS (Version 23; SPSS Inc., Chicago, USA). The results were analyzed by using the Kolmogorov-Smirnov test to determine the normality of the data distribution. Due to the normality of the data, parametric tests were used to analyze. On the other hand, due to the homogeneity of variance test considering that in this study we have 4 independent groups, the parameters were compared by one-way ANOVA, and the LSD test was used as a *post-hoc* test. Differences were considered significant when *P*-values were under 0.05.

## Results

**Figure 1:**
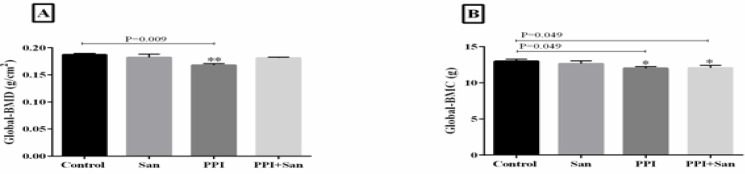
Global bone mineral density and global bone mineral content of the animal models that received different treatments

Bone mineral densitometry data are illustrated in Figure 1 (A & B). The global BMD and BMC of those treated with pantoprazole alone were significantly decreased compared to control groups (*P=*0.009 and *P=*0.049 for BMD and BMC, respectively). There was no significant difference in BMD between the rats that received sandostatin + pantoprazole or sandostatin alone and the control group. 

**Figure 2:**
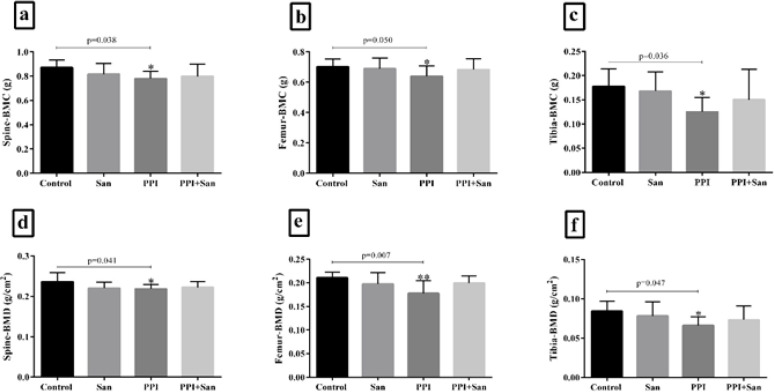
Spine, femur, and tibia bone mineral density and bone mineral content of the animal models that received different treatments

Data analysis showed a significant reduction in BMC and BMD in the femur, tibia, and spine in the PPI group compared to the control group (*P*<0.05). No significant difference was observed among the other groups (Figure 2 a-f).

The serum levels of biochemical parameters in the experimental groups are shown in Table 1. Serum gastrin, PTH, and ALP levels were significantly higher in the pantoprazole group compared to the control group (*P=*0.012, 0.034, and 0.009, respectively) after 90 days. There was no significant difference in the serum levels of gastrin, PTH, and ALP between the rats that received sandostatin + pantoprazole or sandostatin alone and the control group. Although serum calcium and magnesium (Mg) levels were significantly lower in those who received pantoprazole, there was no difference in the serum levels of calcium and Mg between the sandostatin and control groups. 

Table 2 shows the urine biochemical markers. Treatment with pantoprazole alone reduced the urine pH and increased the urinary calcium, phosphorus, chloride, and Mg levels significantly in comparison with other experimental groups (*P*<0.001). Those rats that were treated with sandostatin + pantoprazole had a significantly higher urine pH and lower urinary calcium, phosphorus, chloride, and Mg levels in comparison with those that received pantoprazole alone. 

Figure 1A Global-BMD and Figure 1B Global-BMC

San: Sandostatin group, PPI: proton pump inhibitor (pantoprazole) group, PPI +San: Sandostatin + proton pump inhibitor (pantoprazole) group, *P<0.05, **P<0.01, control vs PPI

Figure 2A: Spine-BMC; Figure 2B Femur-BMC; Figure 2C Tibia-BMC; Figure 2D Spine-BMD; Figure 2E Femur-BMD, Figure 2F Tibia-BMD

San: Sandostatin group, PPI: Proton pump inhibitor (pantoprazole) group, PPI +San: Sandostatin + proton pump inhibitor (pantoprazole) group, *P<0.05, **P<0.01, control vs PPI

**Table 1:** Serum biochemical markers of bone metabolism among different treatment groups of rats

Groups _(n) _	Control _(10)_	San _(10)_	PPI _(8)_	PPI +San _(10)_	F	df	*P*-value
Parameters							
PTH (pg/ml)	64.25±4.43	64.77±3.17	68.36±1.86*	66.71±5.08	2.055	3	0.034
Gastrin (ng/L)	18.22±2.66	19.21±3.66	21.93±3.04*	19.34 ±2.33	2.467	3	0.012
Calcium (mg/dl)	9.71±0.41	9.95±0.45	9.27±0.20*$$$	9.30 ±0.38*$$$	7.075	3	0.021
phosphorus (mg/dl)	5.70±0.47	5.24±0.73	5.46±0.99	5.79 ±0.62	1.199	3	0.482
ALP (IU/L)	261.1±94.5	312.2±89.6	385.6±96.4**	304.6 ±99.9	2.572	3	0.009
Mg^+2^ (mg/dl)	0.49±0.42†††	0.18±0.12†††	1.69±0.56	0.23 ±0.17†††	34.131	3	<0.001

The data are presented as the mean ± SD, *P*<0.05 is considered statistically significant. One-way ANOVA followed by LSD *post hoc*. , n = Numbers of animals in each group

San: Sandostatin, PPI: Proton pump inhibitor (pantoprazole)

*P<0.05, ** *P*<0.01, control vs PPI or PPI +San

$ *P*<0.05, San vs PPI or PPI +San

†††*P*<0.001, PPI vs Control or San or PPI +San

**Table 2:** Urinary calcium, phosphorus, chloride, magnesium, and ph levels (mean ± sd) on the final day of the study in all treated groups of rats

groups _(n) _	control _(10)_	san _(10)_	ppi _(8)_	ppi +san _(10)_	f	df	*P*-value
parameters							
Urinary calcium (mg/day)	0.24±0.08^††^	0.23±0.08^††^	0.35±0.08	0.19±0.07^†††^	8.272	3	<0.001
Urinary phosphorus(mg/day)	2.05±0.90^†††^	2.73±1.01^†^	3.84±1.28	1.87±0.61^†††^	8.285	3	<0.001
Urinary Cl^-^ (mg/day)	2.06±0.95^†††^	1.48±0.53^†††^	3.53±0.96	1.50±0.32^†††^	16.596	3	<0.001
Urinary Mg^+2^ (mg/day)	0.49±0.43^†††^	0.18±0.12^†††^	1.69±0.56	0.23±0.17^†††^	34.131	3	<0.001
Urinary pH	7.57±0.41	8.31±0.25***†††	6.66±0.39***	7.60±0.40†††$$	29.845	3	<0.001

The data are presented as the mean ± SD, *P*<0.05 is considered statistically significant. One-way ANOVA followed by LSD *post hoc*. , n = Numbers of animals in each group

San: Sandostatin, PPI: Proton pump inhibitor (pantoprazole)

†*P*<0.05, †††*P*<0.001, PPI vs Control or San or PPI +San

*P<0.05, control vs PPI or San

$ *P*<0.05, San vs PPI +San

## Discussion

This animal study showed that pantoprazole significantly decreased bone mineral density in rats, but concomitant use of long-acting somatostatin analog prevented bone loss, possibly through inhibition of gastrin secretion. 

The PPIs are widely used for dyspepsia and upper gastrointestinal disorders worldwide (22). Previous investigations revealed that consumption of PPI increased the chance of osteoporotic fracture (23-26). Two main mechanisms were described for osteoporosis induced by PPIs, gastrin over-secretion, and hypochlorhydria (7). Proton pump inhibitors increase the stomach pH by irreversible binding and inhibiting the parietal cells(27). The pH elevation in the stomach enhances gastrin secretion and inhibits the somatostatin secretion(28). Gastrin stimulates the enterochromaffin-like and parietal cells to produce histamine (29). It is shown that histamine accelerates the differentiation of osteoclast precursors and increases bone resorption (30). Furthermore, the increased gastrin level is correlated with PTH over-secretion and, consequently, leads to bone resorption (31). Octreotide is a synthetic somatostatin hormone that inhibits the secretion of many hormones such as gastrin (32). The theory behind this study was that concomitant use of a long-acting octreotide might ameliorate the proton pump inhibitor-induced bone loss by inhibiting gastrin secretion. Interestingly, the results reveal that concomitant use of sandostatin with pantoprazole not only protects the bone, but also ameliorates the changes in biochemical parameters induced by pantoprazole, like increase in serum gastrin, PTH, and alkaline phosphatase. 

Another probable mechanism for PPIs-induced bone loss is hypochlorhydria. Gastric acid suppression reduces the absorption of calcium and magnesium ions from the gastrointestinal tract and causes hypomagnesemia and hypocalcemia that consequently increases serum PTH secretion. Some investigations suggest that octreotide does not have a significant effect on PTH secretion (33). In our study, serum calcium and magnesium levels decreased and serum PTH levels increased among the rats treated with pantoprazole compared to the control group. Concomitant use of sandostatin ameliorated these changes. 

To the best of our knowledge, there is no previous study on the effect of octreotide on bone loss and osteoporosis, specifically or concomitant with a PPI. However, few studies have addressed the effect of octreotide on bone turnover. In an investigation, Clowes *et al*. found that octreotide abolished the glucose-mediated suppression of bone turnover among fifteen healthy subjects. They concluded that the apparent bone turnover response to feeding was probably mediated by an octreotide-inhibitable endocrine factor (34). In an animal study on hypophysectomized rats, GH-stimulated longitudinal bone growth was inhibited by octreotide (35). 

In another animal study on hypophysectomized rats, GH-stimulated longitudinal bone growth was inhibited by octreotide (35). Gagnemo *et al*. found that chickens that received five weeks of omeprazole treatment developed hypergastrinemia, as well as hyperplasia and hypertrophy of the parathyroid glands, associated with increased parathyroid hormone gene expression(36, 37). The femur density of the chickens also reduced significantly (37). A study showed that rats with hypergastrinemia induced by antral exclusion developed hyperparathyroid gland volume and weight due to hyperplasia of the parenchymal cells(38). Our study had some limitations. We did not assess the histology of parathyroid tissues and serum levels of bone resorption markers due to limited funding. 

## Conclusion

This animal study showed that the bone loss and increase in biochemical markers of the bone turnover induced by pantoprazole were preventable by concomitant use of sandostatin, a long-acting somatostatin analog. Despite promising results of concomitant consumption of octreotide with PPIs to prevent osteoporosis, further clinical studies are required.

## Authors’ Contributions

M S was the main investigator for the study and contributed to the design, statistical analysis, and interpretation of data. S T prepared the grant proposal, contributed to the literature search and data collection and analysis, and drafted the paper. F K prepared the rats, assessed all of the bone mineral densities, contributed to data collection and statistical analysis, and drafted the figures. All authors interpreted the data, revised the manuscript for critically important intellectual content, and approved the final version. 

## Conflicts of Interest

The authors declare that there are no conflicts of interest.
